# Antimicrobial stewardship programme implementation in Ho Chi Minh City, Vietnam: a qualitative study to understand healthcare professional behaviours

**DOI:** 10.1186/s13756-026-01798-5

**Published:** 2026-07-28

**Authors:** Phuong Bao Quan, Uyen Thao Uyen Truong, Duy Nguyen Ho, Carmel Hughes, Hoa Quoc Nguyen

**Affiliations:** 1 School of Pharmacy, University of Medicine and Pharmacy at Ho Chi Minh City, 41 Dinh Tien Hoang Street, Sai Gon Ward, Ho Chi Minh City, 70000 Vietnam; 2 School of Pharmacy, Queen’s University Belfast, Belfast, BT9 7BL UK

**Keywords:** Antimicrobial stewardship programme, Behavioral determinants, Healthcare professionals

## Abstract

**Background:**

Implementing antimicrobial stewardship programmes (ASPs) is essential for optimising antimicrobial use and reducing resistance. However, integrating these programmes into Vietnamese healthcare settings remains challenging. This study aimed to explore the behavioral determinants influencing the ASP implementation among healthcare professionals (HCPs) in hospitals in Ho Chi Minh City (HCMC), Vietnam.

**Methods:**

A qualitative study was conducted from April to June 2024 using semi-structured interviews with physicians and clinical pharmacists with at least one year of ASP implementation experience. The interview guide and data analysis were informed by the Behaviour Change Wheel (BCW) and the Theoretical Domains Framework-14 (TDF-14) to identify key mediators affecting ASP practices, which were then mapped onto the COM-B model within the BCW to inform the subsequent intervention development process.

**Results:**

A total of 41 interviews were completed across 12 hospitals. ASP implementation was found to be a multifaceted behaviour influenced by a wide range of interrelated factors. Facilitators included strong professional commitment, accessible clinical guidelines, and collaborative work culture. However, implementation was frequently constrained by non-localised or conflicting guidelines, excessive workloads, insufficient training, and clinical pharmacists’ limited decision-making authority. Identified factors were subsequently mapped to the 14 domains of the TDF-14, with nine domains prioritised for future intervention: “Knowledge”, “Skills”, “Social/professional role and identity”, “Beliefs about capabilities”, “Beliefs about consequences”, “Memory, attention and decision processes”, “Environmental context and resources”, “Social influences”, and “Behavioural regulation”. These nine domains were mapped to the corresponding components of the COM-B model within the BCW, to guide future intervention development.

**Conclusions:**

This study offers theory-informed insights into the behavioral factors affecting the ASP implementation among HCPs, offering a foundation for developing context-specific interventions to improve ASP practices. Future work will focus on mapping key BCW components to behaviour change techniques to design targeted interventions aimed at enhancing ASP effectiveness across Vietnamese hospitals.

**Supplementary Information:**

The online version contains supplementary material available at https://doi.org/10.1186/s13756-026-01798-5.

## Introduction

Antimicrobials, especially antibiotics, are among the most frequently prescribed drug classes worldwide due to their efficacy in preventing and treating infectious diseases. However, their widespread misuse and overuse have considerably accelerated the emergence of antimicrobial resistance (AMR), posing a significant threat to global public health [1]. This has led to reduced efficacy of commonly used antibiotics against infections [2]. In 2019, it was estimated that approximately 4.95 million deaths were associated with AMR globally, Murray et al. [3] highlighting its status as one of the top ten global public health threats facing humanity [4].

Notably, Vietnam ranks amongst the countries with the highest AMR rates in Asia. According to the Asian Network for Surveillance of Resistant Pathogens, Vietnam recorded the highest resistance rates to penicillin (92.0%) and erythromycin (92.1%) amongst 11 surveyed Asian countries [5]. Moreover, the prevalence of multi-drug resistant (MDR) pathogens in Vietnam exceeds that of many neighboring countries. Findings from the Survey of Antibiotic Resistance (SOAR) further revealed that AMR rates amongst *Streptococcus pneumoniae* and *Haemophilus influenzae* isolates in Vietnam were higher than those in Cambodia, Singapore and the Philippines [6].

In response to the growing AMR crisis, many countries have developed national action plans to optimise antimicrobial use. In 2015, the Global Action Plan on Antimicrobial Resistance, endorsed by the World Health Organization (WHO), recommended the implementation of antimicrobial stewardship programmes (ASPs) in healthcare facilities to improve clinical outcomes, reduce antimicrobial misuse and mitigate AMR [7]. Numerous studies have demonstrated that ASPs can significantly improve prescribing behaviours amongst healthcare professionals (HCPs), promoting more appropriate, effective and cost-effective practices [8–11]. However, translating these established benefits into routine practice has proven uneven across health systems, with implementation outcomes shaped heavily by the contexts in which ASPs are introduced.

Implementation outcomes are shaped not only by technical readiness but also by sociocultural and political conditions that generate conflicts and trade-offs amongst the stakeholders involved. Hierarchical professional dynamics, informal prescribing norms, contested professional authority and patient and institutional pressures have been consistently identified as influences on ASP uptake across both high-income and low- and middle-income settings [12–16]. In low- and middle-income countries (LMICs) in particular, additional structural constraints, including limited microbiology capacity, inconsistent regulatory enforcement and workforce shortages, further complicate the conditions under which ASPs can operate [13, 17].

Vietnam’s Ministry of Health issued national guidance for hospital ASP implementation in 2016, with an updated version released in 2020 [18]. The guidance requires hospitals at all tiers, not only tertiary institutions, to establish a hospital-level ASP committee and implement a structured ASP. ASP performance is also incorporated into the national hospital quality assessment framework [18]. Despite these policy advances, ASP implementation has faced persistent challenges. Of the 655 hospitals nationwide, only 144 have successfully implemented ASPs [19]. Furthermore, while some of these hospitals have reported improved antibiotic use, the degree of improvement varies, and few have met all ASP standards [20–22]. These figures suggest that, despite a clear national policy framework, on-the-ground implementation across Vietnamese hospitals remains uneven and incomplete. Available evidence points to leadership engagement, the legitimacy of pharmacist input within physician-led teams, patient and family expectations, and variation between hospital tiers as potential influences on ASP delivery in Vietnam [23–25]. The behavioural mechanisms through which such factors influence HCPs’ day-to-day prescribing and stewardship practice, however, remain poorly characterised.

Given that one of the main components that determine the success of an ASP is active engagement and adherence of HCPs, who are central to antimicrobial prescribing decisions, [12, 13] this study was conducted to understand mediators that influence HCPs’ behaviours in order to tailor effective behaviour change interventions to enhance ASP effectiveness in Ho Chi Minh city (HCMC), Vietnam—a major medical hub with a high patient volumes, diverse healthcare settings, and numerous hospitals where ASPs have been implemented for several years but continue to encounter significant challenges [23].

## Methods

### Study design and participants

This study was conducted from April to June 2024 with physicians and clinical pharmacists from hospitals in HCMC who were actively engaged in clinical practice and had at least one year of experience in implementing ASPs, as these two professional groups are most directly involved in antimicrobial prescribing decisions, prescription review, prescriber feedback, and day-to-day stewardship coordination in the Vietnamese context [18, 26]. HCMC has approximately 162 hospitals across central, city/provincial, district, military, and private sectors, including both general and specialised institutions [27]. Sampling was conducted at the individual healthcare professional level rather than the institutional level. Within this participant-level recruitment approach, maximum-variation sampling was used to ensure representation across settings. Participants were recruited through two main strategies:

- Leveraging the researchers’ network: A list of potential participants was compiled based on the research team’s connections. These participants were typically individuals with deep insights into ASP implementation within their respective hospitals, serving as initial contacts.
- Snowball sampling: Referrals were obtained from recruited participants. Upon completing the interviews, participants were asked to recommend others within their networks who met the study criteria.

### Data collection

Semi-structured interviews were conducted by the researcher (P.B.Q) either in person or via a video call depending on each participant’s preference. Prior to the interviews, written informed consent was obtained and participants were informed that the interviews would be audio-recorded for subsequent analysis.

**Fig. 1:**
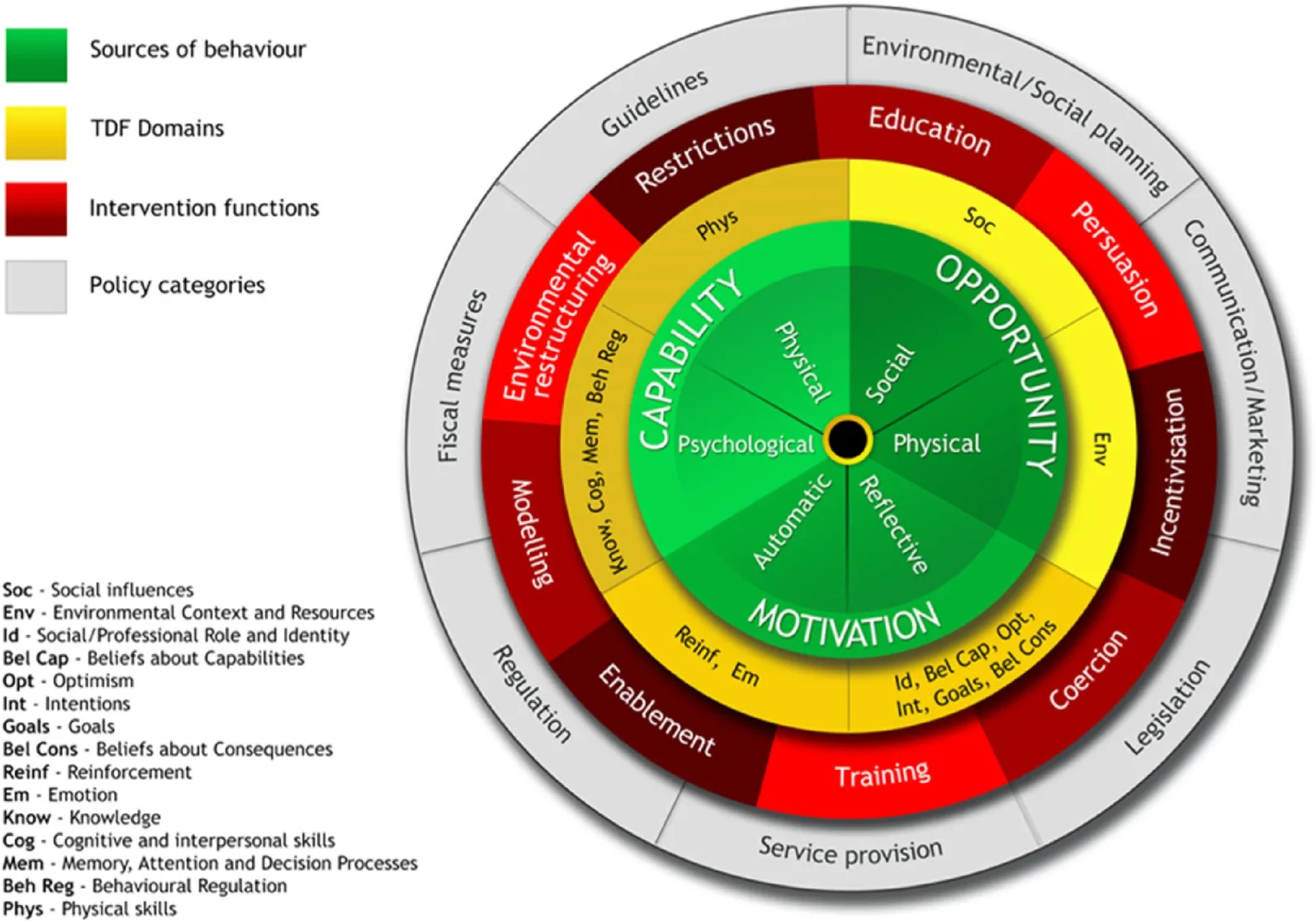
Components of the behaviour change wheel with the integration of TDF-14 [29]

The interviews aimed to explore factors influencing HCPs’ behaviours in ASP practice, including perceived barriers, facilitators and necessary conditions for effective implementation. The topic guide was developed using the Behaviour Change Wheel (BCW), an integrative framework designed to translate behavioural determinants into targeted interventions [28]. At the core of the BCW is the COM-B model, which conceptualises behaviour as the outcome of three interacting components: Capability, Opportunity and Motivation, surrounded by nine intervention functions and seven policy categories that guide intervention design [28]. To enable a more granular analysis, the BCW was integrated with the Theoretical Domains Framework, version 14 (TDF-14), which expands the three COM-B components into 14 behavioural domains capturing the determinants of behaviour change in detail (Fig. 1) [29]. This combined framework allows mediators to be characterised at the domain level and, through systematic examination across all BCW and TDF-14 domains, mapped to the most appropriate behaviour change techniques for subsequent intervention development [29]. Full descriptions of the BCW and TDF-14 frameworks are provided in Supplementary S1.

The guide was structured around four key areas: (1) the current status of ASP implementation at the participant’s hospital; (2) the participant’s perceived role and responsibilities in ASP; (3) perceived facilitators and barriers to ASP implementation; and (4) individual goals and future plans related to ASP. Demographic and professional characteristics, including age, gender, years of experience, and specialty, were systematically collected. Full details of the interview guide are presented in Supplementary S2. While all core topics were consistently covered, specific questions and prompts were adapted in real-time to suit the natural flow of the conversation and the participant’s responses, ensuring contextual relevance and depth. Before fieldwork, two pilot interviews were conducted with volunteer clinical pharmacists who were not included in the main study to assess the clarity, terminology, sequence, and feasibility of the topic guide. These refinements improved the clarity and feasibility of the interview guide for the Vietnamese clinical context but did not alter the underlying study design, theoretical framework, sampling strategy, or analytical approach.

### Data analysis

The data analysis process was conducted through two stages:

- Stage 1: Identifying barriers and facilitators to ASP implementation among HCPs.
- Stage 2: Identifying key TDF domains to be targeted by an intervention

All interview recordings were initially transcribed verbatim in Vietnamese and analysed in their original language to preserve contextual meaning. Transcripts were checked for accuracy, de-identified, and coded anonymously to distinguish physicians (e.g., Physician 1, Physician 2) and clinical pharmacists (e.g., Pharmacist 1, Pharmacist 2). Data collection and analysis were conducted simultaneously until data saturation was achieved—that is, when no new themes or patterns emerged from subsequent interviews [30]. Saturation was treated as an emergent criterion rather than a predefined recruitment endpoint. Recruitment continued until two consecutive interviews generated no new sub-themes within the 14 TDF domains or new inductive themes outside the framework, followed by two additional interviews to confirm saturation and reduce the risk of premature closure.

A combination of deductive and inductive approaches was employed to ensure a comprehensive understanding of the data. Deductive coding was guided by the TDF-14 and COM-B model to identify behavioural mediators relevant to ASP implementation. Inductive thematic analysis was conducted in parrallel on the same transcripts to capture additional emergent factors beyond those encompassed by both the TDF-14 and COM-B model. Each transcript was independently double-coded by two of three researchers (P.B.Q., U.T.U.T., D.N.H.), with one researcher applying the deductive approach and the other applying the inductive approach to the same transcript. To reduce bias and enhance analytical rigor, coding assignments were rotated approximately every 4—5 days so that each researcher applied both approaches across different transcripts over the course of the study. Discrepancies between deductive and inductive codings, or between researchers, were discussed and resolved through consensus discussions with a fourth researcher (H.Q.N.).

In Stage 2, the research team reviewed the coded data to identify key TDF domains for intervention development. Domains were prioritised based on the following criteria: (1) high frequency of coded content; (2) presence of conflicting or diverse perspectives; and (3) strong thematic relevance to ASP-related behaviours [31]. Practical considerations—such as intervention feasibility within hospital or pharmacy settings, resource availability, and project timeline—were also taken into account. Final domain selection was determined through a consensus-based approach among the research team. After finalising the TDF-14 domains to be targeted in a future intervention, they were mapped to the COM-B model within the Behaviour Change Wheel framework to help identify suitable intervention functions and policy categories (Fig. 1). The intervention development process informed by this mapping is beyond the scope of the current paper.

Following the Vietnamese-language analysis, the lead researcher (P.B.Q.) translated all relevant materials—including transcripts, codes, and results—verbatim into English. The translated dataset was then reviewed and proofread by a social science expert to ensure accuracy and clarification. This study also followed the Consolidated Criteria for Reporting Qualitative Research (COREQ) to ensure transparency and methodological rigor (Supplementary S3) [32].

### Triangulation

To enhance the depth and validity of the findings, investigator and methodological triangulation were employed [33]. For investigator triangulation, three researchers (P.B.Q., U.T.U.T., D.N.H.) independently coded each transcript, and a fourth researcher (H.Q.N.) joined consensus discussions to resolve discrepancies, reducing individual interpretive bias. For methodological triangulation, deductive coding guided by TDF-14/COM-B was conducted alongside inductive bottom-up coding of the same transcripts, as described in the Data analysis subsection. This combined approach provided theoretical coverage while allowing unanticipated factors to emerge, addressing limitations of either approach alone [33]. Although not a form of triangulation, the diversity of participants—physicians and clinical pharmacists drawn from 12 hospitals across central, provincial and district tiers and a range of specialties—reflected a deliberate maximum-variation sampling strategy that further supported the credibility and contextual breadth of the findings.

### Ethical consideration

This study was approved by the Research Ethical Committee at the University of Medicine and Pharmacy at HCMC (No. 589/HDDD-DHYD). Participant information was kept confidential in a password-protected laptop and was deleted after the study was completed.

## Results

### Demographics and characteristics of the participants

A total of 50 HCPs from 12 hospitals were invited to participate in the interviews, of whom 9 either declined or did not respond. Data saturation was achieved after 39 interviews; however, two additional interviews were conducted to ensure all potential themes and subthemes were captured, resulting in a total of 41 interviews. Of the 41 participants, 7 were recruited through the researchers’ professional networks, while the remaining 34 were identified via snowball sampling.

Among the participants, 23 (56.0%) were pharmacists and 18 (44.0%) were physicians, representing 12 hospitals across HCMC—including 11 public hospitals (n = 37 participants) and 1 private hospital (n = 4 participants). Of these hospitals, 7 were general and 5 were specialised facilities. Most participants were female (61.0%) and had between 1 and 5 years of professional experience (75.6%). The median interview duration was 30 (interquartile range: 18–37) minutes, with interviews ranging from 13 to 54 min. Detailed participant characteristics are presented in Table 1.

**Table 1:** Characteristics of the interview participants (N=41)

Characteristics		N (%)
Gender	Male	16 (39.0%)
	Female	25 (61.0%)
Age	24**–**29	24 (58.5%)
	30**–**35	10 (24.5%)
	>35	07 (17.0%)
Years of experience	1**–**5 years	31 (75.6%)
	>5 years	10 (24.4%)
Professional title	Physician	18 (43.9%)
	Pharmacist	23 (56.1%)
Specialty	Clinical pharmacy	23 (56.1%)
	Internal medicine	14 (34.2%)
	Surgery	01 (2.4%)
	Pediatrics	03 (7.3%)
Workplace	Public hospital	37 (90.2%)
	Private hospital	04 (9.8%)
Interview length (minutes)	30 (interquartile range: 18–37)	

### Factors associated with the implementation of ASPs by HCPs

The implementation of ASPs by HCPs was shaped by a combination of interrelated factors mapped across the TDF-14 domains. Thematic analysis revealed frequent overlap across domains, with several barriers and facilitators to ASP implementation interacting across multiple TDF-14 domains as described below. The facilitators identified during the analysis are summarised in Table 2 (Section “Facilitators”), and the barriers in Table 3 (Section “Barriers”), each grouped by the corresponding TDF-14 domain. Full TDF-14 domain definitions are provided in Supplementary S1, and the COM-B groupings into which these domains feed are illustrated in Fig. 1.

**Table 2:** Reported themes/subthemes regarding facilitators associated with ASP implementation by TDF-14 domain

TDF-14 domain	Theme/subthemes^(a)^		Representative quotation
Knowledge	Guidelines as a basis for implementation		*“These guidelines serve as the foundation for making recommendations to doctors… It provides stronger justification and greater persuasiveness.”—Pharmacist 1*
	Awareness of ASP		*“Our hospital’s ASP currently follows the Ministry of Health’s guidelines, meaning that we approve antibiotic requests based on the official framework.”—Physician 19*
	Knowledge of treatment guidelines		*“I typically refer to recommendations from IDSA, the Ministry of Health, or the Hospital for Tropical Diseases.”—Physician 4*
Skills	Soft skills	Communication; Researching information	*“One of the most important skills for clinical pharmacists is communication. Pharmacists interact with all stakeholders involved in antibiotic management and dispensing.”—Pharmacist 10*
	Clinical skills	Clinical assessment; Interpreting laboratory tests	*“The first skill is clinical assessment—accurately diagnosing the disease to prescribe the correct antibiotics and assessing disease severity to choose the most appropriate treatment.”—Physician 8*
Social/professional role and identity	ASP as a core responsibility		*“Participating in ASP is one of the key responsibilities of a clinical pharmacist. We were hired to do this, so I see it as a mandatory part of our job.”—Pharmacist 3*
Beliefs about capabilities	Understanding the workflow		*“Since we’ve been doing this for a while, everyone is familiar with the routine tasks.”—Pharmacist 11*
	Confidence in clinical decisions		*“With the tools I have at hand, I feel confident in making clinical decisions.”—Pharmacist 17*
Beliefs about consequences	Training enhances awareness		*“We must emphasize the importance and consequences of inappropriate antibiotic prescriptions. We need to repeatedly remind treating doctors of the burden this issue brings.”—Physician 13*
Reinforcement	Periodic performance evaluation		*“Support should include statistical reports and assessments of antibiotic use effectiveness, patient response, and changes in AMR trends when antibiotics are strictly managed. It’s like a session where we sit together and evaluate our ASP.”—Physician 7*
Environmental context and resources	IT supports implementation		*“The more we integrate IT solutions into the monitoring process, the more we can save manpower and time, leading to more effective implementation.”—Pharmacist 16*
Social influences	Support	Leadership team	*“For any program to run smoothly, strong leadership from senior management or even department heads is essential.”—Pharmacist 11*
		Colleagues	
	Collaboration between departments		*“…we’ve moved from merely signing off prescriptions to now requiring prior antibiotic approval before use. Thanks to the trust we’ve built with physicians, clinical pharmacists are frequently involved in consultations.”—Pharmacist 1*
Behavioural regulation	Implementation planning		*“I create my own slides and assess patient factors when they are admitted, adjusting antibiotic use based on severity.”—Physician 18*

*TDF-14 domains within each section are listed in canonical order. Full domain definitions and constructs are provided in Supplementary S1 (Table 3). Quotations are illustrative and have been translated verbatim from Vietnamese

^(a)^Each theme may relate to multiple TDF-14 domains; in these tables, themes are categorized according to the primary domain

**Table 3:** Reported themes/subthemes regarding barriers associated with ASP implementation by TDF-14 domain

TDF-14 domain	Theme/subthemes^(a)^		Quote
Knowledge	Knowledge discrepancies among HCPs		*“In Vietnam, infectious disease knowledge among pharmacists isn’t standardized yet. In the future, this needs to be addressed to ensure uniform antibiotic expertise.”—Pharmacist 5*
Social/professional role and identity	Lack of awareness of individual role		*“I’ve heard of ASP, but only the name. I don’t really understand what it entails.”—Physician 3*
	Not the final decision-maker		*“In many cases, we provide interventions, but the physician does not agree with our recommendations. In such situations, we can only offer advice and suggestions, while the ultimate decision still rests with the physician who prescribes the treatment.”—Pharmacist 15*
Social/professional role and identity	Limited participation of HCPs		*“…many departments still follow their old ways. They don’t update their practices or adjust their prescribing decisions according to the management guidelines, so overall, there is little improvement.”—Physician 9*
Beliefs about capabilities	Knowledge gaps	Pathology	*“As doctors, pharmacokinetics and pharmacodynamics aren’t our strongest areas—we don’t have the deepest understanding of these aspects.”—Physician 7*
		Microbiology	
		Antibiotics	
	Challenges in antibiotic selection	Emergency cases	*“Patients with multiple comorbidities but mild infection symptoms make treatment decisions difficult. Should we use aggressive therapy or not? Because they’re at higher risk of worsening.”—Physician 16*
		Unclear diagnosis	
		Rare infections	
		Comorbidities	
		Poor clinical response	
		AMR	
	Predominantly empirical treatment		*“Most antibiotic use is still based on experience rather than evidence-based guidelines…”—Physician 1*
	Disagreements with guidelines		*“As a treating physician, I see how severe the patient’s condition is. Even if there’s not enough theoretical justification, I may still choose a stronger antibiotic…”—Physician 7*
	Limited clinical experience		*“Since our clinical pharmacy team is still quite small, and our colleagues don’t have much experience yet, I think we are still not fully equipped to carry out the ASP implementation effectively.”—Pharmacist 2*
	Incomprehensive assessment		*“A patient may need broad-spectrum antibiotics or a stronger pharmacokinetic regimen, but clinical pharmacists rely solely on machine-generated parameters and paperwork without directly seeing the patient. This limits the ability to fully assess the clinical reality, which may result in recommending a milder antibiotic.”—Physician 3*
Beliefs about consequences	Hesitation in antibiotic use	Legal responsibility	*“As a prescribing doctor, I bear legal responsibility for my treatment orders. If they’re incorrect, I could face legal consequences.”—Physician 8*
		Fear of reprimand	
		Bureaucratic procedures	
		Treatment cost	
Reinforcement	Lack of incentives or penalties		*“Currently, there aren’t any concrete measures in place. Our hospital’s ASP has only recently been properly implemented, with clear protocols, probably within the past one or two years.”—Physician 7*
Environmental context and resources	Guidelines	Not practical for local context	*“Most recommendations come from foreign sources, based on populations abroad, with different epidemiological conditions and bacterial environments compared to Vietnam. Even among hospitals, there are differences… so using one hospital’s protocol may not be suitable for another.”—Physician 4*
		Lack of consistency	
	Overloaded workload		*“When conducting consultations and documenting reports, I spend about 30–45 min… pharmacists had to be heavily involved, including a lot of paperwork.”—Pharmacist 5*
	Time constraints		*“The challenge is that we don’t have time to read, yet patients need immediate treatment. In cases like sepsis, antibiotics must be administered within the first hour, and honestly, we don’t have time to think much—we just follow a predefined framework.”—Physician 18*
	Staff shortages		*“Our human resources are quite limited, so we’re not yet able to monitor all departments effectively.”—Pharmacist 16*
	Inefficient IT systems		*“Our hospital’s IT system has quite a few issues. To put it simply, we have to go through a lot of unnecessary and complicated steps.”—Physician 10*
	Limited drug supply		*“The challenge for us is not accessing information but rather the lack of available medications. The guidelines recommend many new drugs that we don’t have.”—Pharmacist 5*
	ASP training limitations		*“There are seminars on treating infectious diseases in hospitals, but there’s still a lack of training on how to manage antibiotic use appropriately.”—Pharmacist 1*
Social influences	Limited communication among HCPs		*“It would be better if clinical pharmacists worked directly in the wards alongside doctors, discussing cases in real time rather than relying on reports and numerical data.”—Physician 3*
	Differences in opinions		*“According to doctors, clinical pharmacists often play the ‘villain’ role—when they want to prescribe drug A, we recommend drug B. So, there will always be some level of conflict between us and the physicians.”—Pharmacist 23*
	Senior HCPs’ points of view		*“Those with more experience tend to have stronger egos and do not value the recommendations from the antimicrobial stewardship program as much.”—Physician 3*
	Patients’ expectations		*“If a doctor assesses that an infection is viral or that antibiotics are unnecessary, patients often feel dissatisfied. They expect to be prescribed antibiotics to feel reassured…”—Physician 8*
Emotion	Discomfort in using antibiotics against personal judgment		*“…in those situations, I feel uncomfortable or pressured because I was unable to successfully communicate my concerns or intervene in the patient’s antibiotic use in time.”—Pharmacist 16*

*TDF-14 domains within each section are listed in canonical order. Full domain definitions and constructs are provided in Supplementary S1 (Table 3). Quotations are illustrative and have been translated verbatim from Vietnamese

^(a)^Each theme may relate to multiple TDF-14 domains; in these tables, themes are categorized according to the primary domain

### Facilitators

All participants indicated that the resources available at their hospitals significantly facilitated ASP implementation. The availability of numerous references—from hospital-level to national and internation guidelines—provided essential guidance for HCPs in executing both clinical practice and ASP-related tasks. In addition, supportive hospital policies, such as regular staff training and the integration of technology into clinical practice, enhanced HCPs’ awareness and work efficiency. Furthermore, regular assessments of ASP implementation at each department were appreciated by the participants as they promoted harmonised implementation across the hospital and improved adherence among HCPs.

“We [clinical pharmacists] visited each clinical department and reviewed about 10 medical records to assess appropriateness of antibiotics use. We then reported the findings in regular hospital meetings, highlighting any issues to ensure healthcare staff were informed and could adjust their practices accordingly.” (Pharmacist 11).

Most participants recognised the benefits of ASP in improving treatment effectiveness and reducing AMR. While all clinical pharmacists demonstrated comprehensive understanding of ASP regulations and procedures, most physicians were generally aware of them but lacked in-depth familiarity. All participants were also well aware of the current treatment guidelines, and believed that effective ASP implementation required honing clinical skills, ranging from diagnostic skills to data interpretation for physicians, communication and continuous professional development for clinical pharmacists. Additionally, interprofessional communication emerged as a vital facilitator, particularly for pharmacists working closely with clinical physicians.

“Since managing antibiotic use requires regular communication with doctors, I need to have good communication skills to talk and discuss with them, and ensure they trust me and follow the treatment guidelines properly.” (Pharmacist 11).

Most participants considered ASP as a routine part of professional responsibilities, and felt confident in their clinical decisions, and actively set goals to optimize antibiotic use*.* They pursued these goals through structured planning, continuous learning, and collaboration on treatment strategies.

Social dynamics also positively influenced ASP efforts. Learning from senior colleagues helped build confidence and reinforce a shared culture of stewardship. Additionally, active leadership support and interdepartmental cooperation were seen to foster morale and streamline coordination.

“As a treating physician, I’m primarily responsible for prescribing antibiotics. My role is to propose the intended treatment, and clinical pharmacists or more experienced colleagues will support by refining those suggestions to better suit the clinical context and align with current guidelines…” (Physician 07).

### Barriers

Despite these facilitators, several challenges constrained ASP implementation. Several HCPs noted that current antimicrobial guidelines—largely based on international data—did not always align with Vietnam’s epidemiological and clinical realities, leading to challenges in application. Discrepancies between guidelines added further uncertainty, making it difficult for HCPs to determine the most appropriate approach.

“Every guideline has some specified clinical features for its country or some guidelines are more up—to-date than the others. That’s why the consistency between these guidelines is vague, there’s no 100% consensus…” (Pharmacist 06).

Workload and time pressure were also common barriers reported by the participants. While the clinical pharmacists found themselves overwhelmed by the number of indicators which required monitoring when patients received antimicrobial therapy, the physicians described their workflows as complex and time-consuming.

“The requirement to obtain approval for each stage makes the whole process very time-consuming, from deciding to use antibiotics to receiving final approval… Managing this process becomes nearly impossible.” (Physician 01).

Limited resources at hospital, including medication and staffing shortages, and inadequate information technology (IT) systems, further hindered effective ASP implementation. Although training programmes helped improve understanding and expedite decision-making, many were inconsistent or insufficient, particularly for physicians. Moreover, in some hospitals, the absence of formal adherence-monitoring mechanisms such as structured audit-feedback systems, incentives, or sanctions linked to ASP compliance further reduced adherence to ASP protocols.

Emotional and motivational barriers were also apparent. Some participants felt their contributions were undervalued by colleagues and supervisors, and many clinical pharmacists expressed the need for formal sanctions to improve compliance. Additionally, although extensive reference materials were available at the institutional level, gaps in knowledge—particilarly in pathology, medical microbiology, and antibiotic use—hampered integration of ASP practices into daily routines, while communication challenges amongst HCPs required additional time and effort to implement ASP. Participants also reported feeling anxious and uncertain when making appropriate choices in complex cases, such as those involving rare conditions, acute infections, or AMR cases presenting with vague symptoms or poor treatment response.

“The pressure comes from cases where a patient is newly admitted, their clinical condition is severe, and I’m uncertain about which antibiotic group to choose or how to combine them. The second challenge is when a patient is already treated with antibiotics, but their clinical condition doesn’t improve, leaving me unsure about how to adjust the antibiotics.” (Physician 09).

Concerns about legal liability, bureaucratic hurdles, and the fear of reprimand discouraged physicians from prescribing “last-resort” antibiotics. Prescribing these antibiotics involves multiple additional steps, including detailed documentation and multi-level approval processes, which are time-consuming and effort-intensive, often deterring physicians from using these drugs even when clinically appropriate. Additionally, several physicians reported limited familiarity with their specific ASP responsibilities. Without a clear understanding of these roles, they tended to rely on personal clinical experience rather than formal guidelines, resulting in inconsistent treatment plans, particularly when multiple physicians were involved in a patient’s care.

“Honestly, using antibiotics based on personal experience is quite common, and since everyone’s experience differs, continuing antibiotic treatment can be challenging. If a patient is treated by one doctor, it’s straightforward, but if multiple doctors are involved, there can be inconsistency—that’s just how it is.” (Physician 12).

On the other hand, clinical pharmacists reported limited clinical experience as a barrier to optimising antibiotic use. Most participants, including physicians, often perceived the assessments of clinical pharmacists as overly guideline-driven and lacking depth, which diminished the value of their recommendations. Moreover, several clinical pharmacists noted their roles were limited to antibiotic recommendations, without having the final decision. Thus, they expressed frustration when their professional assessments were not reflected in treatment decisions.

“The doctor is ultimately responsible for the patient’s legal records, that’s why the clinical pharmacists’ recommendations are only considered supportive and advisory. If doctors don’t agree with my suggestions, they still have the right to make decisions based on their own judgment. In those situations, I tend to feel frustrated…” (Pharmacist 16).

Some respondents also pointed to unprofessional behaviors among colleagues, such as inconsistent prescribing and poor teamwork, which disrupted care coordination. Hierarchical dynamics also shaped clinical decisions, often silencing junior staff (*Social influences*).

“If everyone follows my instructions and decisions, then those who come after will also do the same. If I’m the first to use a particular antibiotic, others will follow my lead, so if I make a mistake, everyone else will too.” (Physician 05).

Patient-related factors had significant influence on HCPs’ antibiotic choices. In some cases, patient expectations and demands for antibiotics—despite clinical inappropriateness—added further complexity to prescribing decisions. Additionally, in Vietnam, many antibiotics are not covered or are only partially covered by national health insurance [34]. Thus, physicians sometimes opted for lower-cost, less effective options due to affordability concerns.

“When patients cannot afford high-efficacy drugs they really need, I am forced to prescribe less effective alternatives, which creates pressure on me.” (Physician 01).

### Identification of key TDF domains to be targeted by an intervention

All 14 domains of the Theoretical Domains Framework (TDF) were found to be relevant to ASP implementation among healthcare professionals. However, through iterative analysis, nine domains were prioritised for intervention design: “Knowledge”, “Skills”, “Social/professional role and identity”, “Beliefs about Capabilities”, “Beliefs about Consequences”, “Memory, attention and decision processes”, “Environmental context and resources”, “Social influences” and “Behavioural regulation”. Five domains—“Reinforcement”, “Emotion”, “Goals”, “Intentions”, and “Optimism”—were not prioritised for intervention. While themes related to “Reinforcement” and “Emotion” were present, addressing them would require longer-term, resource-intensive interventions. Similarly, “Goals” and “Intentions” were commonly reported as individual-dependent internal factors amongst participants, and “Optimism” overlapped with other domains, making it less actionable as a standalone focus.

A detailed mapping of the mediators to both the TDF-14 and BCW domains influencing ASP implementation is provided in Supplementary S4. All three components of the COM-B model were represented in the prioritised TDF domains: Capability (“Knowledge”, “Skills”, “Memory, attention and decision processes”, “Behavioural regulation”), Opportunity (“Environmental context and resources” and “Social influences”), and Motivation (“Social/professional role and identity”, “Beliefs about Capabilities”, “Beliefs about Consequences”). These components were subsequently used to guide the selection of appropriate intervention functions and policy categories within the BCW framework to support intervention development. The detailed design of these interventions lies beyond the scope of the present paper.

## Discussion

This study addresses a critical gap in the existing literature by examining ASP implementation among HCPs from behavioural perspectives. Our findings revealed that ASP practices were shaped by a complex interplay of facilitators and barriers spanning multiple behavioural domains, with overlapping influences often mapped across the TDF.

Among the 14 domains, “Environmental context and resources” and “Knowledge” were particularly prominent and identified as key intervention targets. The participants emphasised the crucial roles of hospital resources in integrating ASP into their daily routines. Clear, diverse, and easily accessible guidelines provided essential support for guiding antibiotic use, promoting rational antibiotic prescribing, and ensuring consistency among HCPs. Similar findings were reported by Talpaert et al*.* [35], who noted that treatment guideline development contributed to reducing the use of third-generation cephalosporins and fluoroquinolones while maintaining treatment efficacy. However, participants in our study encountered difficulties in applying these guidelines into their daily practices, as they were often developed based on research conducted in other countries. This challenge aligned with a 2021 nationwide study across 16 Vietnamese hospitals, which identified the lack of locally relevant guildelines as a major barriers to ASP implementation [24]. These findings underscore the need for evidence-based guidelines tailored to the Vietnamese context to standardise treatment and improve patient care quality.

Heavy workload and time pressure further hindered ASP-related tasks, as staff shortages compelled HCPs to juggle multiple responsibilities, thereby increasing workloads and reducing task efficacy [14, 36]. Additionally, complex procedures and limited IT systems exacerbated these issues by prolonging antibiotic prescribing processes, especially for antibiotics requiring prior approval. While pre-authorisation processes encouraged cautious antibiotic use and reduced overprescribing, they also disrupted workflows and added time pressure [37, 38]. Consequently, some physicians resorted to prescribing lower-efficacy antibiotics to circumvent these administrative barriers.

The domain of “Social/professional role and identity” also emerged as central to ASP implementation. While clinical pharmacists had a clear understanding of their roles, most physicians lacked full awareness of their responsibilities in implementing ASPs. The participants reported that hospital training sessions focused on updating practice guidelines and introducing new treatments, with limited emphasis on ASP-specific training. This emphasises the importance of tailored training programmes to enhance ASP awareness and effective implementation among HCPs [24, 39]. Physicians were also noted to adhere poorly to guidelines, often relying on personal experience rather than evidence-based practices. This finding is consistent with previous studies reporting that physicians’ skepticism about guideline effectiveness contributes to poor adherence [40, 41]. Additionally, antibiotic selection by physicians was influenced by multiple factors, including disease severity, patient affordability and demand [25]. Several physicians in the study tended to prescribe high-efficacy antibiotics for acute or life-threatening cases. Therefore, it is important that antibiotic decision-support tools are developed to enhance evidence-based prescribing practices.

The “Skills” domain highlighted the necessity for healthcare professionals to possess not only clinical expertise but also cognitive and interpersonal skills to effectively carry out ASP-related responsibilities. Although clinical experience was deemed crucial, several clinical pharmacists had limited exposure to hands-on clinical practice. This gap might stem from current pharmacy education programmes, which prioritise theoretical training but lack sufficient practical components for applying these concepts in clinical settings [42]. Furthermore, clinical pharmacists’ roles were often limited to providing recommendations on antibiotic use rather than directly managing treatment, leading to delays in implementing necessary interventions. Empowering clinical pharmacists with greater authority and supportive policies could improve treatment quality and minimise errors in antibiotic use [43].

“Social influences” also played a significant role in shaping stewardship behaviours. Supportive policies from hospital boards facillitated ASP adoption among HCPs by providing training and effectively allocating resources [14, 40]. Collaboration among colleagues boosted morale and enhanced adherence to treatment protocols. Previous studies, such as those by Alkhamees et al. [44] and Baraka et al*. *[14] have similarly demonstrated the benefits of interprofesional collaboration in improving treatment adherence and achieving concensus in decision-making. However, conflicts occasionally arose due to senior HCPs’ reluctance to accept recommendations from junior staff, [15, 17] emphasising the need for training programmes to improve communication, align treatment approaches, and foster mutual respect. Aditionally, patient demands for unnecessary antibiotics posed significant challenges, [16, 45] underscoring the importance of patient education in reducing inappropriate antibiotic use and enhancing treatment outcomes.

All 14 TDF domains were found to be relevant to ASP implementation as they were frequently coded during data analysis and reflected a range of barriers and facilitators impacting HCP behaviours. However, not all domains were considered feasible or necessary to target within a future intervention. Nine domains were identified as ‘key domains’ for future intervention development based on the frequency and salience of themes, potential for behaviour change, and practical considerations regarding implementation in the hospital setting. These findings, grounded in theory and practice, provide a strong foundation for designing a context-specific, behaviourally informed intervention to strengthen ASP implementation among HCPs in Vietnam.

This study is the first in Vietnam to explore HCPs’ behaviours in ASP implementation using a theoretical framework approach. In-depth interviews, based on the integration of TDF-14 and the COM-B model within BCW, identified key mediators influencing ASP implementation. These findings offer practical direction for designing interventions that can meaningfully support ASP implementation in hospital settings. The study also involved a diverse sample of HCPs from multiple hospitals in HCMC, offering a comprehensive and multidimensional perspective on the current state of ASP implementation. However, the use of individual interviews may have limited the findings, as participants may have been reluctant to share views openly due to fear of judgement, being constrained by time, appeared eager to conclude quickly and provided limited recall or elaboration on specific experiences. Additionally, although the focus on physicians and clinical pharmacists was a deliberate scoping choice intended to reflect their central operational roles in current Vietnamese ASP practice (see Methods), the perspectives of other stakeholders, including infection control, nurses and microbiologists, were not captured directly. As the roles of these professional groups within Vietnamese ASPs continue to develop, expanded research that includes them will be an essential next step. Future studies should therefore extend the present analysis to these groups, as well as include participants from various regions and healthcare settings across Vietnam, to provide a more comprehensive multidisciplinary understanding of ASP implementation nationwide.

## Conclusion

This study provided valuable insights into ASP implementation and its associated factors among HCPs. While the value of ASPs were widely recognised, HCPs encountered numerous challenges into their daily practices. By applying the TDF, nine key behavioural domains were identified and mapped across the three components of the COM-B model within the BCW, highlighting priority areas for future intervention. Further research will focus on mapping the identified components to behaviour change techniques to inform the development of a theory-driven intervention aimed at strengthening ASP implementation across hospitals in Vietnam.

## Supplementary Information

Below is the link to the electronic supplementary material.

Supplementary Material 1.

Supplementary Material 2.

Supplementary Material 3.

Supplementary Material 4.

## Data Availability

Study data is avalaible from the corresponding author upon request.
