# Supplementary material for: Antimicrobial stewardship programme implementation in Ho Chi Minh City, Vietnam: a qualitative study to understand healthcare professional behaviours
Source: Antimicrob Resist Infect Control. 2026 Jul 28;15:132. doi: 10.1186/s13756-026-01798-5 (PMC13700437; doi:10.1186/s13756-026-01798-5)
Supplement: Supplementary file 1 [file 13756_2026_1798_MOESM1_ESM.docx]

**Supplementary S1. Description of the Behaviour Change Wheel and Theoretical Domain Framework-14 (TDF-14)**

**1. Definitions of interventions and policies according to Behavior Change Wheel**

| **Interventions** | **Definition** | **Examples** |
| --- | --- | --- |
| Education | Increasing knowledge or understanding | Providing information to promote healthy eating |
| Persuasion | Using communication to induce positive or negative feelings or stimulate action | Using imagery to motivate increases in physical activity |
| Incentivisation | Creating expectation of reward | Using prize draws to induce attempts to stop smoking |
| Coercion | Creating expectation of punishment or cost | Raising the financial cost to reduce excessive alcohol consumption |
| Training | Imparting skills | Advanced driver training to increase safe driving |
| Restriction | Using rules to reduce the opportunity to engage in the target behaviour (or to increase the target behaviour by reducing the opportunity to engage in competing behaviours) | Prohibiting sales of solvents to people under 18 to reduce use for intoxication |
| Environmental restructuring | Changing the physical or social context | Providing on-screen prompts for GPs to ask about smoking behaviour |
| Modelling | Providing an example for people to aspire to or imitate | Using TV drama scenes involving safe-sex practices to increase condom use |
| Enablement | Increasing means/reducing barriers to increase capability or opportunity | Behavioural support for smoking cessation, medication for cognitive deficits, surgery to reduce obesity, prostheses to promote physical activity |
| **Policies** | | |
| Communication/marketing | Using print, electronic, telephonic or broadcast media | Conducting mass media campaigns |
| Guidelines | Creating documents that recommend or mandate practice. This includes all changes to service provision | Producing and disseminating treatment protocols |
| Fiscal | Using the tax system to reduce or increase the financial cost | Increasing duty or increasing anti-smuggling activities |
| Regulation | Establishing rules or principles of behaviour or practice | Establishing voluntary agreements on advertising |
| Legislation | Making or changing laws | Prohibiting sale or use |
| Environmental/social planning | Designing and/or controlling the physical or social environment | Using town planning |
| Service provision | Delivering a service | Establishing support services in workplaces, communities etc. |

**2. Definitions and component constructs of TDF-14**

| **Domain (definition)** | **Constructs** |
| --- | --- |
| 1. Knowledge  (An awareness of the existence of something) | Knowledge (including knowledge of condition/scientific rationale)  Procedural knowledge  Knowledge of task environment |
| 2. Skills  (An ability or proficiency acquired through practice) | Skills  Skills development  Competence  Ability  Interpersonal skills  Practice  Skill assessment |
| 3. Social/professional role and identity  (A coherent set of behaviours and displayed personal qualities of an individual in a social or work setting) | Professional identity  Professional role  Social identity  Identity  Professional boundaries  Professional confidence  Group identity  Leadership  Organisational commitment |
| 4. Beliefs about capabilities  (Acceptance of the truth, reality or validity about an ability, talent or facility that a person can put to constructive use) | Self-confidence  Perceived competence  Self-efficacy  Perceived behavioural control  Beliefs  Self-esteem  Empowerment  Professional confidence |
| 5. Optimism  (The confidence that things will happen for the best or that desired goals will be attained) | Optimism  Pessimism  Unrealistic optimism  Identity |
| 6. Beliefs about Consequences  (Acceptance of the truth, reality, or validity about outcomes of a behaviour in a given situation) | Beliefs  Outcome expectancies  Characteristics of outcome expectancies  Anticipated regret  Consequents |
| 7. Reinforcement  (Increasing the probability of a response by arranging a dependent relationship, or contingency, between the response and a given stimulus) | Rewards (proximal/distal, valued/not valued, probable/improbable)  Incentives  Punishment  Consequents  Reinforcement  Contingencies  Sanctions |
| 8. Intentions  (A conscious decision to perform a behaviour or a resolve to act in a certain way) | Stability of intentions  Stages of change model  Transtheoretical model and stages of change |
| 9. Goals  (Mental representations of outcomes or end states that an individual wants to achieve) | Goals (distal/proximal)  Goal priority  Goal/target setting  Goals (autonomous/controlled)  Action planning  Implementation intention |
| 10. Memory, attention and decision processes  (The ability to retain information, focus selectively on aspects of the environment and choose between two or more alternatives) | Memory  Attention  Attention control  Decision making  Cognitive overload/tiredness |
| 11. Environmental context and resources  (Any circumstance of a person’s situation or environment that discourages or encourages the development of skills and abilities, independence, social competence and adaptive behaviour) | Environmental stressors  Resources/material resources  Organisational culture/climate  Salient events/critical incidents  Person × environment interaction  Barriers and facilitators |
| 12. Social influences  (Those interpersonal processes that can cause individuals to change their thoughts, feelings, or behaviours) | Social pressure  Social norms  Group conformity  Social comparisons  Group norms  Social support  Power  Intergroup conflict  Alienation  Group identity  Modelling |
| 13. Emotion  (A complex reaction pattern, involving experiential, behavioural, and physiological elements, by which the individual attempts to deal with a personally significant matter or event) | Fear  Anxiety  Affect  Stress  Depression  Positive/negative affect  Burn-out |
| 14. Behavioural regulation  (Anything aimed at managing or changing objectively observed or measured actions) | Self-monitoring  Breaking habit  Action planning |
