# Supplementary material for: Antimicrobial stewardship programme implementation in Ho Chi Minh City, Vietnam: a qualitative study to understand healthcare professional behaviours
Source: Antimicrob Resist Infect Control. 2026 Jul 28;15:132. doi: 10.1186/s13756-026-01798-5 (PMC13700437; doi:10.1186/s13756-026-01798-5)
Supplement: Supplementary file 2 [file 13756_2026_1798_MOESM2_ESM.docx]

**Supplementary S2. Interview guide**

**I. Introduction**

Good morning/afternoon,

First of all, we sincerely appreciate your willingness to participate in today’s interview. The purpose of this interview is to explore your thoughts and perceptions regarding the implementation of the Antimicrobial Stewardship Program (ASP) in your hospital. The information collected will help assess the factors influencing healthcare professionals' execution of ASP. Based on these insights, our research team aims to propose interventions to enhance ASP implementation in hospitals.

Today’s interview will last approximately 30–40 minutes. Before we begin, please take a moment to read the research information sheet.

[Provide the participant with the informed consent form]

Do you have any questions regarding the research? If not, please sign the informed consent form. Signing this form indicates that you voluntarily agree to participate in the study. You may withdraw at any time, and all information you provide will be kept confidential in accordance with research ethics guidelines.

Once you have signed the consent form, we will begin the interview. To facilitate later analysis, we seek your permission to record this interview. We assure you that all information shared today will remain confidential, and the recording will be deleted immediately after the study concludes. Do you consent to the recording?

During the interview, to ensure confidentiality, please avoid mentioning your own name or the names of any individuals related to your responses.

[Start recording]

**II. General Information**

1. What year were you born?
2. Is your hospital a public or private institution?
3. Which department do you work in? What is your current position?

**III. ASP Implementation in the Hospital**

4. Are you involved in ASP implementation at your hospital? How long have you been engaged in ASP activities?
5. How is ASP implemented in your hospital?
   - When was ASP introduced at your hospital? Who is involved in the implementation? What are the main activities?
   - Who are the members of the ASP management team in your hospital? How is it organised?
   - In your opinion, how effective is ASP in optimizing antimicrobial use for patients? What specific improvements have you observed (e.g., antimicrobial selection, dosage, duration, antimicrobial resistance rates, etc.)? – **Goals, Optimism**
6. Have you heard of Decision No. 5631/QD-BYT on "Guidelines for Antimicrobial Stewardship in Hospitals"? If so, how do you understand its principles? – **Knowledge**
   - How do the ASP guidelines at your hospital compare to the Ministry of Health’s guidelines?
   - What sources do you use to stay updated on new evidence or recommendations related to ASP?
   - In your opinion, what are the benefits and challenges of adhering to ASP guidelines? – **Beliefs about consequences**

**IV. Personal Role in ASP**

7. What is your role in ASP implementation? – **Social/professional role and identity**
   - How do you contribute to ASP execution?
   - Are there any aspects of ASP you feel uncertain about or need to improve to optimise your involvement? – **Knowledge**
   - What challenges do you face when making ASP-related decisions that fall outside your responsibilities?
8. How does ASP implementation affect your workload and workflow? – **Beliefs about consequences**
9. How do you ensure consistent adherence to ASP recommendations and principles? – **Behavioral regulation**
   - Have there been instances where you were unable to follow ASP guidelines?
   - How do you typically address such situations?
   - What measures do you think could enhance adherence to ASP guidelines?
10. Has your hospital developed guidelines, procedures, or support tools for antimicrobial use in clinical practice? – **Memory, attention, and decision processes**
    - If so, how do you access this information?
    - What challenges do you face in accessing and retrieving this information?
    - How could these challenges be overcome?
    - Do you frequently look up ASP-related knowledge during clinical practice?
    - In what situations do you need to refer back to information before making a decision?
11. In your opinion, what skills are essential for physicians/pharmacists to effectively implement ASP? – **Skill**
    - What clinical and soft skills should a physician/pharmacist/nurse possess to execute ASP effectively?
12. How confident do you feel about your ASP-related decisions in clinical practice? (Very confident, confident, slightly confident, not confident) – **Beliefs about capabilities**
    - In what situations do you feel less confident or not confident at all in making decisions?
13. In what scenarios do you feel pressure when making decisions regarding antimicrobial use for patients? – **Emotion**
    - What are the sources of this pressure? (Personal, hospital policies, societal expectations, etc.)
    - How does this pressure affect your ASP implementation?
    - How do colleagues, patients, and other healthcare professionals influence your decision-making? – **Social influences**
14. If you experience discomfort or pressure to prescribe antimicrobials against your professional judgment, how do you handle such situations? – **Emotion**
15. Does your hospital have an evaluation, reward, or disciplinary system related to ASP implementation? – **Reinforcement**
    - If so, how is performance assessed and rewarded/penalised according to hospital policies?
    - Do you feel your efforts are adequately recognised and supported?
    - What supportive policies or incentives would motivate you to follow ASP guidelines and recommendations?

**V. Facilitators and Barriers**

16. What challenges or limitations have you encountered in ASP implementation? – **Skill, Social influences, Environmental context and resources**
    - Apart from the challenges you mentioned, are there any other difficulties in applying ASP in clinical practice?
    - Have you faced difficulties due to societal pressures or existing norms and expectations?
17. What resources would help you implement ASP more effectively in your hospital? – **Beliefs about capabilities, Environmental context and resources**
    - Are these resources currently available in your hospital?
    - If so, do you have any suggestions for improving or optimizing these resources?
18. In your opinion, what aspects of ASP implementation in your hospital need improvement? – **Optimism**
    - What barriers might hinder improvements in ASP implementation?
19. Does your hospital/department regularly conduct ASP training sessions? If so, what topics are covered? – **Skill**
    - Do you find these training sessions helpful? Why or why not?
    - What additional training topics would support you in implementing ASP more effectively?

**VI. Goals and Future Plans**

20. What specific goals have you set for yourself regarding ASP implementation? – **Goals**
    - Do your hospital’s ASP goals align with your personal and professional aspirations?
21. What are your plans to contribute to ASP at your hospital? – **Intentions**
22. How would the introduction of new evidence or hospital policies on antimicrobial use impact your future ASP implementation? – **Intentions**

**VII. Closing the Interview**

This concludes our interview.

Do you have any additional thoughts or concerns related to ASP implementation at your hospital? How do you feel about today’s interview? Was there anything during the interview that made you feel uncomfortable?

Thank you very much for your time and valuable insights today.
