# Supplementary material for: Antimicrobial stewardship programme implementation in Ho Chi Minh City, Vietnam: a qualitative study to understand healthcare professional behaviours
Source: Antimicrob Resist Infect Control. 2026 Jul 28;15:132. doi: 10.1186/s13756-026-01798-5 (PMC13700437; doi:10.1186/s13756-026-01798-5)
Supplement: Supplementary file 4 [file 13756_2026_1798_MOESM4_ESM.docx]

**Supplementary S4. Mapping key factors to TDF-14 and BCW domains**

| **FACILITATORS** | **CAPABILITY** | | | | | **OPPORTUNITY** | | **MOTIVATION** | | | | | | | |
| --- | --- | --- | --- | --- | --- | --- | --- | --- | --- | --- | --- | --- | --- | --- | --- |
|  | **Phys^+^** | **Psychological** | | | | **Social** | **Physical** | **Reflective** | | | | | | **Automatic** | |
|  | Physical skills**^@^** | Knowledge | Cognitive and interpersonal skills**^@^** | Memory, attention and decision processes | Behavioral regulation | Social influences | Environmental context and resources | Beliefs about capabilities | Beliefs about Consequences | Social/professional role and identity | Optimism | Intentions | Goals | Reinforcement | Emotion |
| Extensive reference sources available |  |  |  |  |  |  |  |  |  |  |  |  |  |  |  |
| Guidelines serve as a basis for implementation |  |  |  |  |  |  |  |  |  |  |  |  |  |  |  |
| Enhanced policies to support implementing ASP |  |  |  |  |  |  |  |  |  |  |  |  |  |  |  |
| Training enhances awareness |  |  |  |  |  |  |  |  |  |  |  |  |  |  |  |
| Periodic performance evaluation |  |  |  |  |  |  |  |  |  |  |  |  |  |  |  |
| Awareness of ASP**^g^** |  |  |  |  |  |  |  |  |  |  |  |  |  |  |  |
| Improved communication among HCPs |  |  |  |  |  |  |  |  |  |  |  |  |  |  |  |
| Meticulous ASP implementation planning |  |  |  |  |  |  |  |  |  |  |  |  |  |  |  |
| Competence in clinical assessment |  |  |  |  |  |  |  |  |  |  |  |  |  |  |  |
| Ability to search for and synthesize literature |  |  |  |  |  |  |  |  |  |  |  |  |  |  |  |
| Self-directed learning and experience enhancement |  |  |  |  |  |  |  |  |  |  |  |  |  |  |  |
| ASP as a core responsibility |  |  |  |  |  |  |  |  |  |  |  |  |  |  |  |
| Appropriate treatment selection |  |  |  |  |  |  |  |  |  |  |  |  |  |  |  |
| Confidence in clinical decisions |  |  |  |  |  |  |  |  |  |  |  |  |  |  |  |
| Understanding the workflow |  |  |  |  |  |  |  |  |  |  |  |  |  |  |  |
| Support from colleagues |  |  |  |  |  |  |  |  |  |  |  |  |  |  |  |
| Support from leadership team |  |  |  |  |  |  |  |  |  |  |  |  |  |  |  |
| Strengthened interprofessional collaboration |  |  |  |  |  |  |  |  |  |  |  |  |  |  |  |
| Treatment perspectives of experienced HCPs |  |  |  |  |  |  |  |  |  |  |  |  |  |  |  |
| HCPs’ awareness |  |  |  |  |  |  |  |  |  |  |  |  |  |  |  |
| Coordinated ASP implementation across departments |  |  |  |  |  |  |  |  |  |  |  |  |  |  |  |
| **BARRIERS** | Physical skills **^@^** | Knowledge | Cognitive and interpersonal skills**^@^** | Memory, attention and decision processes | Behavioral regulation | Social influences | Environmental context and resources | Beliefs about capabilities | Beliefs about Consequences | Social/professional role and identity | Optimism | Intentions | Goals | Reinforcement | Emotion |
| Time constraints |  |  |  |  |  |  |  |  |  |  |  |  |  |  |  |
| Limitation in guidelines**^a^** |  |  |  |  |  |  |  |  |  |  |  |  |  |  |  |
| Complex and inconsistent procedures**^b^** |  |  |  |  |  |  |  |  |  |  |  |  |  |  |  |
| Overloaded workload**^c^** |  |  |  |  |  |  |  |  |  |  |  |  |  |  |  |
| Staff shortages |  |  |  |  |  |  |  |  |  |  |  |  |  |  |  |
| Limited drug availability |  |  |  |  |  |  |  |  |  |  |  |  |  |  |  |
| Weak ASP monitoring and evaluation |  |  |  |  |  |  |  |  |  |  |  |  |  |  |  |
| Inadequate ASP training |  |  |  |  |  |  |  |  |  |  |  |  |  |  |  |
| Lack of ASP enforcement measures |  |  |  |  |  |  |  |  |  |  |  |  |  |  |  |
